# Evolutionary Significance of Fungal Hypermutators: Lessons Learned from Clinical Strains and Implications for Fungal Plant Pathogens

**DOI:** 10.1128/msphere.00087-22

**Published:** 2022-05-31

**Authors:** Nikita Gambhir, Steven D. Harris, Sydney E. Everhart

**Affiliations:** a Plant Pathology and Plant-Microbe Biology Section, Cornell University, Geneva, New York, USA; b Department of Plant Pathology and Microbiology; Department of Entomology, Iowa State University, Ames, Iowa, USA; c Department of Plant Science and Landscape Architecture, University of Connecticut, Storrs, Connecticut, USA; University of Georgia

**Keywords:** hypermutator, DNA mismatch repair, MMR defect, MSH2, antifungal resistance, fungicide resistance, adaptation, microevolution, evolution, mutation rate

## Abstract

Rapid evolution of fungal pathogens poses a serious threat to medicine and agriculture. The mutation rate determines the pace of evolution of a fungal pathogen. Hypermutator fungal strains have an elevated mutation rate owing to certain defects such as those in the DNA mismatch repair system. Studies in Saccharomyces cerevisiae show that hypermutators expedite evolution by generating beneficial alleles at a faster pace than the wild-type strains. However, an accumulation of deleterious alleles in a hypermutator may reduce its fitness. The balance between fitness cost and mutation benefit determines the prevalence of hypermutators in a population. This balance is affected by a complex interaction of ploidy, mode of reproduction, population size, and recent population history. Studies in human fungal pathogens like Aspergillus fumigatus, Candida albicans, Candida glabrata, Cryptococcus deuterogattii, and Cryptococcus neoformans have highlighted the importance of hypermutators in host adaptation and development of antifungal resistance. However, a critical examination of hypermutator biology, experimental evolution studies, and epidemiological studies suggests that hypermutators may impact evolutionary investigations. This review aims to integrate the knowledge about biology, experimental evolution, and dynamics of fungal hypermutators to critically examine the evolutionary role of hypermutators in fungal pathogen populations and project implications of hypermutators in the evolution of fungal plant pathogen populations. Understanding the factors determining the emergence and evolution of fungal hypermutators can open a novel avenue of managing rapidly evolving fungal pathogens in medicine and agriculture.

## INTRODUCTION

Mutations can be produced either due to errors in DNA replication or DNA damage by environmental or intrinsic factors. Since many nonsynonymous mutations are likely to be deleterious, organisms have evolved two mutation avoidance mechanisms, proofreading by DNA polymerase and the mismatch repair (MMR) system. Errors generated during DNA replication are first rectified by the proofreading activity of DNA polymerase, which decreases the mutation rate of the organism by 10- to 100-fold (1). The errors that escape proofreading are subjected to MMR, which further reduces the mutation rate by 50- to 1,000-fold (2). Some of the mutations resulting from DNA damage and recombination are also rectified by MMR. But what if these mutation avoidance mechanisms become defective? Studies in bacteria, fungi, and mammalian cancer cells have found that MMR defects confer a hypermutator phenotype with an elevated mutation rate (3–5). Although this phenotype leads to cancer in mammals, it can expedite the evolution of pathogen populations by generating a plethora of mutations for selection to act upon. However, an accumulation of deleterious mutations may reduce its fitness and render this phenotype advantageous for short-term adaptation (6).

Bacterial hypermutators are recognized to hasten the evolution of antibiotic resistance, virulence acquisition, host adaptation, and disease transmissibility (3, 7). The role of hypermutators in fungal pathogen evolution has only gained medical attention in the last decade, while scant attention has been paid to agricultural implications. Studies in laboratory strains of Saccharomyces cerevisiae and human-pathogenic fungi have shown that hypermutators can expedite stress adaptation and mediate antifungal resistance and host adaptation (4, 8, 9). Given the importance of hypermutators, this review will critically examine the studies on biology, experimental evolution, and population dynamics of hypermutator S. cerevisiae and human fungal pathogens to gain a better understanding of the factors shaping the evolutionary trajectories of hypermutators, how hypermutator biology may impact evolutionary investigations, and the agricultural implications of hypermutators. For the sake of brevity, hypermutators arising from MMR defects will be the focus of this review.

## GENETIC BASIS OF HYPERMUTATOR EMERGENCE AND VARIATION IN MUTATION RATE

**FIG 1:**
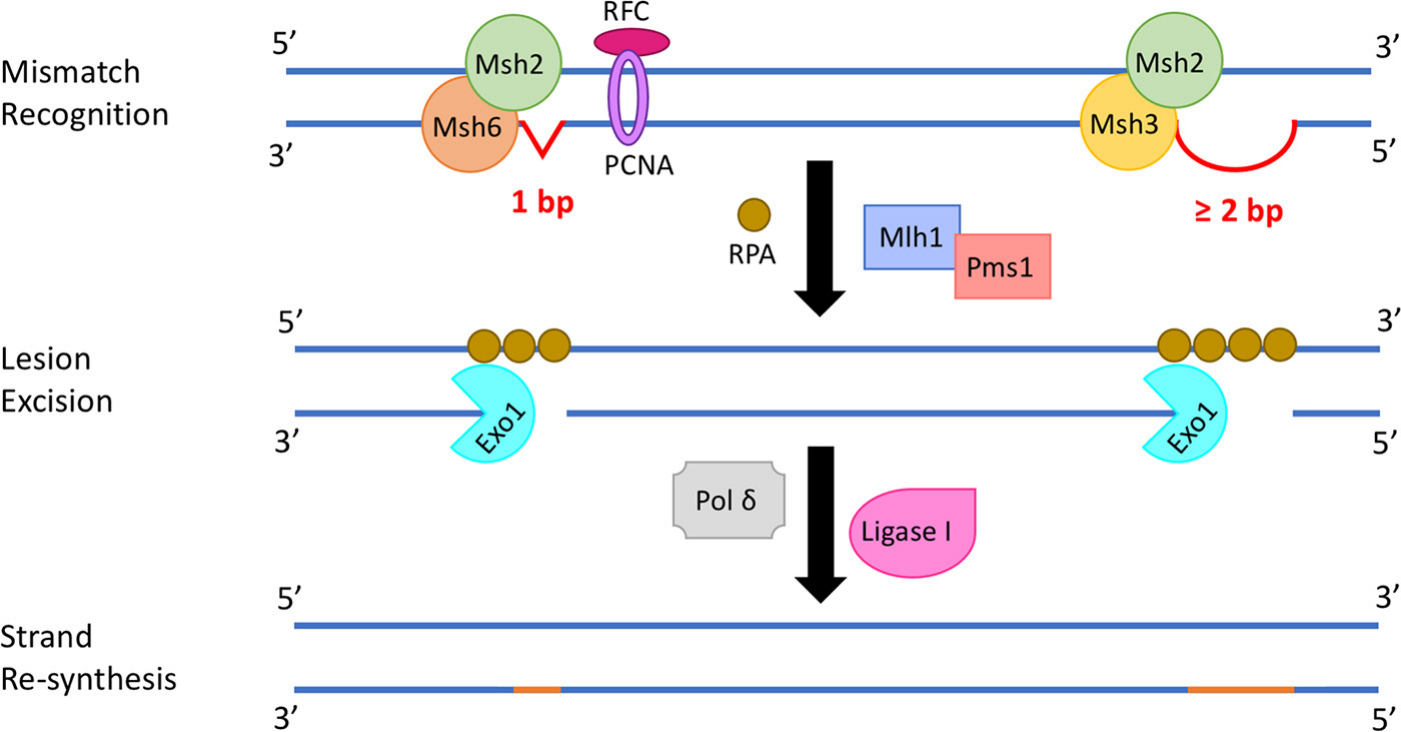
Schematic diagram of the mismatch repair (MMR) pathway that rectifies errors arising from DNA replication in Saccharomyces cerevisiae. Mismatches are recognized by the Msh heterodimers. The Msh2-Msh6 heterodimer primarily identifies base-base and single insertion/deletion (1-bp) mismatches, the Msh2-Msh3 heterodimer primarily identifies longer insertion/deletion loop (≥2-bp) mismatches, and the Mlh1-Pms1 heterodimer directs downstream events. Replication factor C (RFC) loads the proliferating cell nuclear antigen (PCNA), which interacts with various proteins and is involved in multiple steps in the pathway (although it is shown once for simplicity). Lesions in the newly synthesized strand are then excised by exonuclease 1 (Exo1), while the replication protein A (RPA) binds to single-stranded DNA (ssDNA). The DNA polymerase, Pol δ, synthesizes the new strand, and ligase I ligates the fragments of the new strand.

Hypermutators can arise from nonsynonymous mutations in one or more genes involved in the MMR pathway. In Escherichia coli, the MMR system consists of three “Mut” proteins, MutS, MutL, and MutH. While MutS binds to mismatches, MutL integrates mismatch detection with downstream processing, and MutH cleaves the newly synthesized DNA strand for subsequent exonuclease activity (10–14). In S. cerevisiae, multiple homologs of the bacterial Mut proteins are involved in mitotic and meiotic mutation avoidance. While six MutS homologs (Msh1 to Msh6) and four MutL homologs (Mlh1 to Mlh3 and Pms1) have been identified, no homolog of MutH is known (14–17). Among the Msh proteins, Msh1 maintains mitochondrial genomic stability, and other Msh proteins function as heterodimers to maintain nuclear genomic stability. The Msh2-Msh6 heterodimer is primarily involved in repairing base-base and single insertion/deletion mismatches, the Msh2-Msh3 heterodimer primarily repairs longer insertion/deletion loop mismatches (Fig. 1), and the Msh4-Msh5 heterodimer facilitates crossing over during meiosis. The Mlh heterodimers, Mlh1-Pms1, Mlh1-Mlh2, and Mlh1-Mlh3, direct downstream events in mitotic mutation avoidance and meiotic recombination (14, 18).

Nonsynonymous mutations in one or more MMR genes can increase the mutation rate of the fungal strain, conferring a hypermutator phenotype. Considerable variation in the mutation rate of hypermutators has been observed in natural fungal populations (9, 19–21). The mutation rate is determined by three factors, (i) the MMR gene that harbors the nonsynonymous mutation, (ii) the amino acid position affected by the nonsynonymous mutation, and (iii) the strain’s genetic background.

Since MMR genes differ in their functions, the mutation rate of a hypermutator would depend on the defective MMR gene it harbors. Mutations in *MSH2* and *MLH1* genes are more disruptive for the organism than mutations in other MMR genes, as these mutations could disrupt the function of all the heterodimers involved in the MMR pathway (8, 22). Additionally, individual nonsynonymous mutations can exhibit a wild-type mutation rate but can significantly increase the mutation rate when present together. For example, an incompatible combination (or negative epistatic interaction) of certain *MLH1* and *PMS1* alleles (*cMLH1-kPMS1*) can increase the mutation rate of S. cerevisiae up to 340-fold (19, 20).

Different nonsynonymous mutations in the same MMR gene can vary in the mutation rate they confer (4, 8, 9, 21, 23–25). The position of the mutation would determine which motif it affects and to what degree it disrupts the protein’s three-dimensional structure (26, 27). For example, among 54 nonsynonymous mutations in the *MSH2* gene of S. cerevisiae, the increase in mutation rate varied from 1- to 282-fold. About 55% of the mutations conferred high mutation rates, 8% of mutations conferred an intermediate increase in mutation rate, and 38% of mutations showed a wild-type mutation rate (26).

Interestingly, the same nonsynonymous mutation can render different mutation rates in different strain backgrounds owing to the presence of genomic suppressors or enhancers of mutation rate (20, 28–32). For example, the incompatible *cMLH1-kPMS1* combination showed a 196-fold-higher mutation rate in the S288c strain background but showed a wild-type mutation rate in the YJM523 strain background (32).

## MUTATION SPECTRA AND THEIR IMPACT ON EVOLUTIONARY INVESTIGATIONS

A defect in the MMR genes can increase the rate of all types of mutations, single nucleotide polymorphisms (SNPs), insertions/deletions (indels), and structural variants (27, 33, 34). While SNPs are more likely to occur in coding regions with a bias toward higher G-to-A transitions (8, 27, 34), indels are more likely to occur in noncoding regions (27). This mutation profile might not necessarily result from the MMR defect itself, but possibly because coding indels are more likely to be disruptive to protein function than coding SNPs. Mutations in repetitive sequences are the hallmark of MMR defects. Studies in S. cerevisiae, Candida glabrata, Cryptococcus deuterogattii, and Cryptococcus neoformans show that a defective MMR leads to mutations in long homopolymeric nucleotide tracts (8, 9, 27, 33–36) and microsatellites (27, 34, 37). This can be attributed to the inefficacy of DNA polymerase proofreading activity to rectify errors in homopolymer runs of >7 nucleotides long, rendering MMR as the sole machinery repairing such defects (35, 38). Indels in repetitive sequences are more prominent than elsewhere in the genome (27). The mutability of the repetitive sequence increases with its length. A 51,000-fold-increase in mutability was observed in indels in 14-bp-long homopolymer sequences compared to 4-bp-long homopolymer runs (35).

Owing to extensive mutations and rapidly changing mutation profiles, determining evolutionary relationships with hypermutator strains may lead to erratic conclusions. In phylogenetic studies, distantly related hypermutator strains may form a pseudophylogenetic cluster owing to the increased indels in homopolymer runs. This phenomenon is called long-branch attraction (LBA), and parsimony methods are more prone to LBA than likelihood methods. Hence, caution should be exercised while interpreting evolutionary relationships among hypermutators. Additionally, closely related hypermutator strains may appear to be distantly related. In a recent study, C. neoformans isolates were obtained from patients before and after relapse of infection to discern if the infection was caused by the same or a different isolate (39). Sixteen of the 17 pairs of isolates were recurrent pairs. All the recurrent pairs of isolates had ≥97% SNP similarity and clustered together with short branch lengths phylogenetically with the exception of one pair of isolates that had 56% SNP similarity and distinctly long branch lengths. This observation could have led to an interpretation of reinfection by a different isolate, but sequence analysis of the *MSH2*, *MSH5*, and *RAD5* genes revealed that the two isolates were hypermutators, their allele profiles were identical, and the infection was a true relapse and not a reinfection. Hence, to more accurately determine the evolutionary relationships among fungal strains, sequence information of MMR genes can be helpful.

A few studies genotyped *MSH2*-defective C. glabrata strains using microsatellites and/or multilocus sequence typing (MLST) and concluded that *MSH2*-defective alleles can be genotype specific (21, 23, 24). All strains (*n* = 63) belonging to one microsatellite genotype had the V239L mutation in the *MSH2* gene (24). However, two different microsatellite genotypes (Gt22, *n* = 2, and Gt36, *n* = 5) consisted of both the wild-type *MSH2* allele and P208S/N890I mutations (23). Results from microsatellite genotyping are questionable since MMR defects lead to microsatellite instability. When C. glabrata strains were genotyped using MLST, all the strains (*n* = 10) in the sequence type 10 (ST10) genotype had the same P208S/N890I mutation in two different studies (21, 24). In contrast, the V239L mutation was found to be associated with ST7 genotype in one study (*n* = 104) (24) and with the ST8 genotype in another study (*n* = 2) (33). Since homopolymeric runs can occur in several genes (9) used in MLST and mutations can also occur in coding sequences devoid of homopolymer runs, MLST genotyping may be affected by MMR defects. Given that MMR defects lead to extensive genomic mutations, especially in microsatellites and long homopolymeric runs, genetic markers should be carefully chosen for genotyping hypermutator strains.

Although extensive genomic mutations can be deleterious for the fitness of a hypermutator over time, an MMR defect can hitchhike with a beneficial allele and be indirectly selected for short-term adaptation. A balance between fitness cost and mutation benefit determines the prevalence (or frequency) of hypermutators in a population. This balance is further governed by species- and population-specific factors.

## HYPERMUTATOR DYNAMICS IN FUNGAL POPULATIONS

Experimental evolution studies in S. cerevisiae populations have evaluated the mutation benefit and fitness cost of hypermutators and found that results vary with ploidy, mode of reproduction, and population size (40–42). Populations with a fixed ratio of *msh2Δ* strains and wild-type strains were propagated for 100 to 400 generations for mutation accumulation. The final frequency of *msh2Δ* strains indicated if mutation benefit or fitness cost was higher.

The frequency of hypermutators is expected to decline in sexual populations due to a lack of association between the mutator and beneficial alleles owing to recombination. However, a beneficial allele generated by a hypermutator can still propagate in a sexual population and aid in adaptation. In sexual populations of S. cerevisiae, the frequency of hypermutators declined (41). In addition to outcrossing, the decline could have been due to reduced spore viability due to deletion of one MMR gene. Although MMR deletion mutants have reduced spore viability (18, 19, 43, 44), some naturally occurring nonsynonymous mutations in MMR genes do not show such a defect (28).

In asexual populations, mutator alleles can hitchhike with beneficial alleles and increase in frequency. However, the outcome can be affected by ploidy. An increase in ploidy can mask deleterious alleles and be advantageous for adaptation (45, 46). Consistent with this hypothesis, an increased fitness and frequency of hypermutators was observed in diploid asexual populations of S. cerevisiae (40, 41). Hypermutators in haploid asexual populations would be expected to yield more deleterious mutations and lead to a decline in the frequency of the hypermutator strains, but various results have been observed in different population sizes of S. cerevisiae (42). If a beneficial allele emerges earlier in a hypermutator strain, hypermutators would increase in their frequency within the population (47). In small populations (~10^5^ cells) of S. cerevisiae, the mutator allele hitchhiked with the beneficial allele to fixation in 100 generations. With an increase in population size, the mutator allele took longer to hitchhike with the beneficial allele. This delay could have been due to clonal interference, which is a competition between clonal lineages with different beneficial mutations. In large (10^6^ to 10^7^ cells) to very large populations (~10^8^ cells), there is an increased probability of wild type to generate beneficial alleles early on, which decreases the relative benefit of the MMR defect, and hypermutators decrease in frequency (40–42). These experiments suggest that a complex interplay among ploidy, mode of reproduction, and population size may determine the prevalence of hypermutators in a population. It should be noted that these evolutionary trajectories are determined for deletion strains that represent extreme cases. However, mutation rates of hypermutators in natural populations show considerable variation, which may affect their evolutionary trajectories.

The prevalence of nonsynonymous MMR mutations in natural populations varies among and within species. About 13% of A. fumigatus isolates had a nonsynonymous mutation in the *MSH2* homologue (48), 44 to 72% of C. glabrata isolates had a nonsynonymous mutation in the *MSH2* gene (4, 23–25), and 2% of isolates had the incompatible *MLH1* and *PMS1* alleles in S. cerevisiae (32). We hypothesize that such variation in prevalence of nonsynonymous MMR mutations can be explained by the differences in the mode of reproduction of the species. In sexually reproducing A. fumigatus and S. cerevisiae, outcrossing between hypermutators and wild-type strains could have broken the association of mutator and beneficial alleles. Saccharomyces cerevisiae showed less prevalence of MMR defects than A. fumigatus because the probability of three alleles occurring together (one beneficial allele and two incompatible MMR alleles) is lower than two alleles occurring together. Additionally, the differences can possibly be attributed to the dynamics of nuclear cooperation and competition in the multinucleate A. fumigatus. Since only asexual reproduction has been documented in C. glabrata, a higher prevalence of nonsynonymous mutations may suggest that a hypermutator phenotype can be an important mechanism to increase genetic diversity, and the mutation benefit can be higher than the fitness cost in asexual haploid populations.

In a given population, there can be alternating periods of high and low prevalence of hypermutators (49). Even in the absence of recombination, the mutation rate of a population may change over time (50, 51). Fungal pathogens encounter a number of stressors when adapting to the host, like high temperature, hypoxia, unfavorable pH, nutrient deprivation, and reactive oxidative and nitrosative species (52). After successful colonization of the host, pathogens can be exposed to antifungal stress. Under these changing stress conditions, hypermutators can rescue the population by aiding in adaptation. Mutator alleles can frequently emerge in a population, get selected by hitchhiking with beneficial alleles, and help the population to survive a particular stress condition. Over time, hypermutators can decrease in frequency due to negative selection owing to reduced fitness or by emergence of antimutator (or suppressor) alleles. The frequency of hypermutators in a population not only depends on species and population biology but may also depend on the population’s recent history of stress exposure (50).

## ROLE OF HYPERMUTATORS IN ADAPTATION OF HUMAN FUNGAL PATHOGENS

The role of hypermutators in antifungal resistance development and/or within-host adaptation has been investigated in several human pathogens, including Aspergillus fumigatus (48), C. albicans (53), C. glabrata (4, 21, 23–25, 33), C. deuterogattii (9), and C. neoformans (8, 39). While the presence of selection pressure is critical, hypermutators can produce more progeny strains with favorable phenotypes for selection to act upon (4, 8). Pathogens with nonsynonymous MMR mutations were isolated from patients, and MMR genes were deleted from some strains to determine their effect on antifungal resistance and virulence. In C. glabrata, *in vitro* transfers on antifungal-amended media led to an increase in resistance frequency in *msh2Δ* strains by ~82-, 18-, and 9-fold for caspofungin, fluconazole, and amphotericin B, respectively, compared to the wild-type strains. An increased resistance rate to caspofungin was also observed in mouse models. However, when mice were coinfected with both the wild-type and *msh2Δ* strains at a ratio of 1:1, wild-type strains were able to colonize the mouse gut better than the mutants (4). In C. neoformans, *msh2Δ*, *mlh1Δ*, and *pms1Δ* mutants rapidly developed resistance to fluconazole and amphotericin B than the wild-type strains in the presence of the drug. Although *pms1Δ* mutants showed reduced virulence, *msh2Δ* and *mlh1Δ* mutants did not have reduced virulence (8). Wild-type strains have a fitness advantage in favorable conditions or once adaptation has been achieved (9, 32, 40, 54) because an accumulation of deleterious mutations can reduce their virulence (4, 9, 48).

Direct evidence of stress adaptation in clinical MMR-defective strains has been shown by isolating paired samples from patients before and after stress exposure. Nonsynonymous mutations in *MSH2* and *MSH5* genes led to the microevolution of C. neoformans in an HIV-positive patient causing a recurrent infection (39). Microevolution to antifungal drug resistance has also been observed. One pair of C. glabrata strains with a nonsynonymous mutation in the *MSH2* gene was isolated before and after 50 days of fluconazole therapy from an HIV-positive patient (33). Owing to the high selection pressure, the sequential isolate developed azole resistance.

MMR defects have been found in both antifungal-resistant and -susceptible clinical strains of C. glabrata. Nonsynonymous *MSH2* polymorphisms were observed in 42.9% of fluconazole-resistant isolates, 80.6% of fluconazole-sensitive isolates, and 100% of echinocandin-resistant isolates (24). Because of a high prevalence of MMR defective strains and their lack of association with antifungal resistance, the role of hypermutators in antifungal drug resistance has been questioned (23–25). However, this observation can be explained by the variation in selection pressures on MMR-defective strains. Hypermutators can only confer antifungal resistance if they had an antifungal drug exposure. In clinical strains of C. glabrata isolated from France, *MSH2* nonsynonymous polymorphisms were observed in 48% of the isolates with high fluconazole MICs and 42.8% of isolates with low fluconazole MICs (23). When the treatment history for each patient was taken into account, exposure to antifungal drugs was found to be associated with resistance occurrence. Clinical strains of C. glabrata isolated from India had 69% prevalence of MMR-defective strains, but no echinocandin- or azole-resistant strains were found (25). Such an observation may have resulted from a relatively weak selection pressure on the population, as echinocandin treatment was only given to 1% of the patients in the study, and strains were isolated from patients within 2 weeks of azole therapy. Additionally, despite a high prevalence of nonsynonymous MMR mutations, the presence of antimutator alleles could have mitigated the increase in mutation rate.

The high prevalence of MMR-defective strains in asexual C. glabrata populations may reflect the importance of this phenotype to adapt to changing stress conditions in the human body. Since hypermutators can expedite stress adaptation in human fungal pathogens, it is likely that hypermutators may hasten adaptation of fungal pathogens present in other stressful environments like agriculture. Currently, no study has evaluated the role of hypermutators in the evolution of fungal plant pathogens. The following are some implications and considerations for pursuing research on hypermutators in this area.

## CAN HYPERMUTATORS EXPEDITE EVOLUTION IN FUNGAL PLANT PATHOGENS?

In agriculture, the practice of monoculture is prevalent, which means that genetically uniform plants are grown over large acreages. Monoculture exerts a strong selection pressure on pathogen populations for host adaptation. Host adaptation is especially important for obligate biotrophic pathogens, as they can only survive on a living host and are under a high selection pressure to evolve virulence. Biotrophic plant-pathogenic fungi secrete proteins, called effectors, to combat plant defenses and mediate virulence. Effector genes are often located in rapidly evolving compartments of the fungal genome, such as repeat-rich regions (55), and many effector proteins themselves contain repetitive sequences like leucine-rich repeats. Since MMR defects especially increase mutations in repetitive sequences, a hypermutator phenotype could be advantageous in evolving novel effectors.

Fungicide applications also exert a strong selection pressure to develop resistant plant pathogens. Extensive fungicide use has resulted in rapid evolution of resistance in some pathogens. Resistance was reported as early as 2 years after the launch of some fungicides (56). Interestingly, resistance comes at a cost of virulence in some isolates of different plant-pathogenic species (57–60). In Cercospora beticola, 50% of competition experiments between isolates that were sensitive and resistant to demethylation-inhibitor fungicides showed that resistance was associated with reduced spore production and virulence (59). One possible explanation could be that the three-dimensional structure of the mutated protein (encoded by the gene harboring the resistance mutation) may negatively impact important biochemical pathways and may have downstream effects on spore production and virulence. Another likely explanation could be that some isolates with the resistance mutation may be hypermutators, and they may have accumulated mutations in genes important for spore production and virulence.

Experimental and epidemiological studies are required to assess the role of hypermutators in stress adaptation of plant pathogens. Currently, MMR genes have not been experimentally validated in plant-pathogenic fungi, but genome sequencing and transcriptomic projects in several pathogens, including Fusarium verticillioides, have identified putative genes involved in the MMR pathway (61, 62). Additionally, different strains of the same species have been found to have variable mutation rates, but the mechanism behind such variation has not yet been explored (63). More recently, comprehensive phylogenomic characterization of MMR pathways in ascomycete fungi revealed broad conservation across the phylum (64). A notable exception is the significant loss of MMR capacity in the powdery mildew genera *Erysiphe* and *Blumeria*, which correlates with accelerated genome evolution.

Mutation accumulation experiments can be conducted for validating the putative MMR genes. However, mutation accumulation studies in plant pathogens will be different from those conducted in S. cerevisiae, as most of the plant-pathogenic fungi are strictly filamentous. In filamentous fungi, cells are not discrete entities but are connected to each other to form hyphae. This may combine mutations from different nuclei and cause rapid accumulation of mutations (65), decreasing the likelihood of emergence of a hypermutator phenotype. However, a recent study in the filamentous human fungal pathogen A. fumigatus suggests that hypermutators can confer an adaptive advantage under stress (48). Thus, filamentous growth may still permit the emergence of hypermutators. Moreover, certain features of filamentous fungal growth may be advantageous for hypermutators. For example, heterokaryosis is a common phenomenon in filamentous fungi in which two or more genetically distinct nuclei are present in the same cell. Heterokaryosis may facilitate the coexistence of nuclei with and without MMR defects in the same cell, which may confer the mutation benefit without the fitness cost (at least transiently). In the rice sheath blight pathogen, Rhizoctonia solani, a heterokaryon containing both the wild-type and fungicide resistance alleles conferred a resistance phenotype (66). On repeated exposure to the thifluzamide fungicide, the heterokaryon evolved into a homokaryon with resistance alleles in all nuclei. Thus, filamentous fungal growth of plant-pathogenic fungi may allow shifts in the proportion of MMR defective and wild-type nuclei within cells in response to changes in the selection pressure, which may be advantageous for hypermutator-mediated adaptation.

## NON-MMR FORMS OF HYPERMUTATION

In addition to MMR, other DNA damage response pathways have also been linked to hypermutator effects in fungi (67). For example, recombination caused by replication stress or attempts to repair damaged bases increases the risk of genome rearrangements such as translocations and other chromosomal aberrations. Detailed studies in yeast have shed light on the pathways and complexes that both prevent and promote genome rearrangements (68). In the filamentous fungi, use of model systems such as Aspergillus nidulans and Neurospora crassa has provided some insight into processes involved in preventing genome instability and hypermutation (69, 70). As in yeast, the response to replication stress is likely to play a prominent role in triggering instability, though there is also evidence that distinct mutation avoidance mechanisms might exist in filamentous fungi (62, 67). Furthermore, an additional consideration is that many filamentous fungal plant pathogens possess repeat-rich “pathogenicity chromosomes” that are particularly susceptible to recombination events that lead to genome instability (67). Although less is known about these pathways than MMR, their potential importance merits further investigation of their roles in promoting hypermutation.

## CONCLUSION AND FUTURE DIRECTIONS

**FIG 2:**
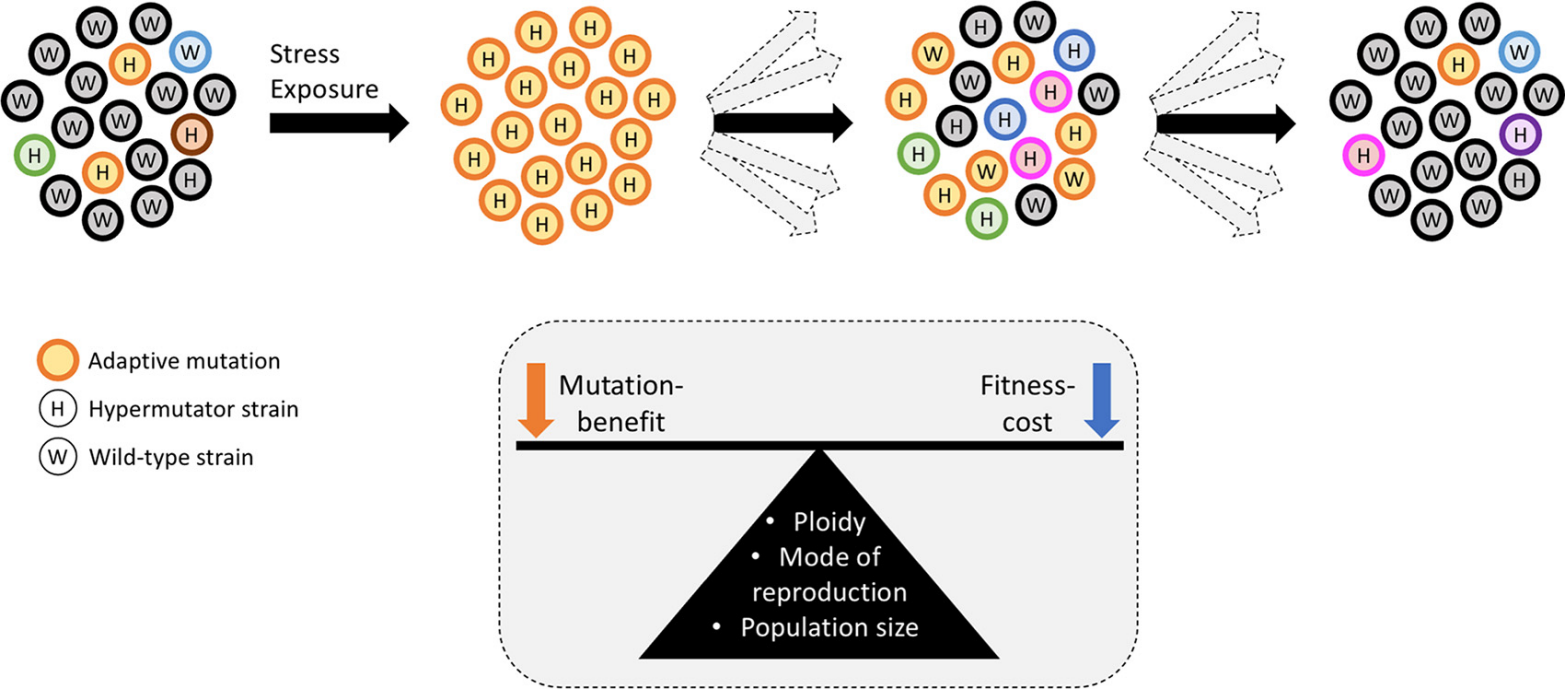
Proposed trajectory of hypermutator prevalence in a fungal population over time. Hypermutator (H) strains can generate more genetic diversity (represented by different colors) than the wild-type (W) strains in a population. On stress exposure, and when H strains have the adaptive mutation (orange outline), the hypermutator allele can hitchhike with the adaptive mutation, resulting in stress adaptation of the population and an inadvertent increase in the prevalence of H. When the stress is removed, there can be multiple evolutionary trajectories (represented by gray arrows) depending on the balance of mutation benefit and fitness cost, which is further governed by factors such as ploidy, mode of reproduction, and population size. Shown is one possible evolutionary trajectory where in the absence of selection pressure, some H strains may start losing the association of the adaptive mutation and hypermutator allele (due to recombination with a migrated W strain), or the wild-type mutation rate may be restored in H due to emergence of antimutator alleles. At this initial phase of stress removal, H can be more prevalent than W, and hence, the population has high genetic variability. Over time, H may accumulate a high number of mutations; some of which may occur in important housekeeping or virulence genes, which can reduce the fitness of H strains, and consequently, the prevalence of H can decrease.

Hypermutators can expedite antifungal resistance and host adaptation in human fungal pathogens, thus rescuing populations from stress. However, such a phenotype may not be beneficial in long-term adaptation, which is conceptually represented in Fig. 2. The frequency of hypermutators in a population is determined by an interaction of ploidy, mode of reproduction, population size, and its recent population history. Although hypermutators facilitate evolution, their rapidly changing mutation profiles may render them unreliable in determining their evolutionary relationships with other strains. Knowledge gained from S. cerevisiae and human fungal pathogens can be applied in plant pathogens to enhance our understanding about the role of hypermutators in fungicide resistance development and host adaptation.

A limitation of the majority of studies on hypermutators is that they mainly focus on the *MSH2* gene. Although it is one of the major genes involved in the MMR pathway, further research is required to understand the role of other MMR genes in evolution of hypermutators. Moreover, it will be important to determine how MMR is integrated with other mechanisms that trigger mutator phenotypes in fungal plant pathogens (67). Additionally, identification of biochemical targets of antimutator alleles is required. These alleles have been found to modulate the phenotype of MMR defects (20, 28–32). The YJM523 strain of S. cerevisiae was homozygous for *cMLH1-kPMS1* incompatibility but still conferred a wild-type phenotype, owing to antimutator alleles present in the genome (32). Knowledge of biochemical pathways used by antimutator alleles to suppress the hypermutator phenotype can be used to design novel drugs to mitigate the evolution of fungal hypermutators in medicine and agriculture.

## References

[1] Manhart CM, Alani E. 2017. DNA replication and mismatch repair safeguard against metabolic imbalances. Proc Natl Acad Sci USA 114:5561–5563. doi:10.1073/pnas.1705971114. 28533419 PMC5465931

[2] Iyer RR, Pluciennik A, Burdett V, Modrich PL. 2006. DNA mismatch repair: functions and mechanisms. Chem Rev 106:302–323. doi:10.1021/cr0404794. 16464007

[3] Oliver A, Mena A. 2010. Bacterial hypermutation in cystic fibrosis, not only for antibiotic resistance. Clin Microbiol Infect 16:798–808. doi:10.1111/j.1469-0691.2010.03250.x. 20880409

[4] Healey KR, Zhao Y, Perez WB, Lockhart SR, Sobel JD, Farmakiotis D, Kontoyiannis DP, Sanglard D, Taj-Aldeen SJ, Alexander BD, Jimenez-Ortigosa C, Shor E, Perlin DS. 2016. Prevalent mutator genotype identified in fungal pathogen *Candida glabrata* promotes multi-drug resistance. Nat Commun 7:11128. doi:10.1038/ncomms11128. 27020939 PMC5603725

[5] Peltomäki P. Role of DNA mismatch repair defects in the pathogenesis of human cancer. 2003. J Clin Oncol 21:1174–1179. doi:10.1200/JCO.2003.04.060. 12637487

[6] Denamur E, Matic I. Evolution of mutation rates in bacteria. 2006. Mol Microbiol 60:820–827. doi:10.1111/j.1365-2958.2006.05150.x. 16677295

[7] Chopra I, O'Neill AJ, Miller K. 2003. The role of mutators in the emergence of antibiotic-resistant bacteria. Drug Resist Updat 6:137–145. doi:10.1016/S1368-7646(03)00041-4. 12860461

[8] Boyce KJ, Wang Y, Verma S, Shakya VPS, Xue C, Idnurm A. 2017. Mismatch repair of DNA replication errors contributes to microevolution in the pathogenic fungus *Cryptococcus neoformans*. mBio 8:e00595-17. doi:10.1128/mBio.00595-17. 28559486 PMC5449657

[9] Billmyre RB, Blake BR, Clancey SA, Heitman J. 2017. Natural mismatch repair mutations mediate phenotypic diversity and drug resistance in *Cryptococcus deuterogattii*. Elife 6:e28802. doi:10.7554/eLife.28802. 28948913 PMC5614558

[10] Su SS, Modrich P. 1986. *Escherichia coli* mutS-encoded protein binds to mismatched DNA base pairs. Proc Natl Acad Sci USA 83:5057–5061. doi:10.1073/pnas.83.14.5057. 3014530 PMC323889

[11] Grilley M, Welsh KM, Su SS, Modrich P. 1989. Isolation and characterization of the *Escherichia coli* mutL gene product. J Biol Chem 264:1000–1004. doi:10.1016/S0021-9258(19)85043-3. 2536011

[12] Au KG, Welsh K, Modrich P. 1992. Initiation of methyl-directed mismatch repair. J Biol Chem 267:12142–12148. doi:10.1016/S0021-9258(19)49816-5. 1601880

[13] Modrich P, Lahue R. 1996. Mismatch repair in replication fidelity, genetic recombination, and cancer biology. Annu Rev Biochem 65:101–133. doi:10.1146/annurev.bi.65.070196.000533. 8811176

[14] Boiteux S, Jinks-Robertson S. 2013. DNA repair mechanisms and the bypass of DNA damage in *Saccharomyces cerevisiae*. Genetics 193:1025–1064. doi:10.1534/genetics.112.145219. 23547164 PMC3606085

[15] Reenan RA, Kolodner RD. 1992. Isolation and characterization of two *Saccharomyces cerevisiae* genes encoding homologs of the bacterial HexA and MutS mismatch repair proteins. Genetics 132:963–973. doi:10.1093/genetics/132.4.963. 1459447 PMC1205252

[16] New L, Liu K, Crouse GF. 1993. The yeast gene *MSH3* defines a new class of eukaryotic MutS homologues. Mol Gen Genet 239:97–108. doi:10.1007/BF00281607. 8510668

[17] Marsischky GT, Filosi N, Kane MF, Kolodner R. 1996. Redundancy of *Saccharomyces cerevisiae MSH3* and *MSH6* in *MSH2*-dependent mismatch repair. Genes Dev 10:407–420. doi:10.1101/gad.10.4.407. 8600025

[18] Prolla TA, Christie DM, Liskay RM. 1994. Dual requirement in yeast DNA mismatch repair for *MLH1* and *PMS1*, two homologs of the bacterial mutL gene. Mol Cell Biol 14:407–415. doi:10.1128/mcb.14.1.407-415.1994. 8264608 PMC358390

[19] Heck JA, Argueso JL, Gemici Z, Reeves RG, Bernard A, Aquadro CF, Alani E. 2006. Negative epistasis between natural variants of the *Saccharomyces cerevisiae MLH1* and *PMS1* genes results in a defect in mismatch repair. Proc Natl Acad Sci USA 103:3256–3261. doi:10.1073/pnas.0510998103. 16492773 PMC1413905

[20] Raghavan V, Bui DT, Al-Sweel N, Friedrich A, Schacherer J, Aquadro CF, Alani E. 2018. Incompatibilities in mismatch repair genes *MLH1-PMS1* contribute to a wide range of mutation rates in human isolates of baker’s yeast. Genetics 210:1253–1266. doi:10.1534/genetics.118.301550. 30348651 PMC6283166

[21] Healey KR, Ortigosa CJ, Shor E, Perlin DS. 2016. Genetic drivers of multidrug resistance in *Candida glabrata*. Front Microbiol 7:1995. doi:10.3389/fmicb.2016.01995. 28018323 PMC5156712

[22] Fishel R. 2001. The selection for mismatch repair defects in hereditary nonpolyposis colorectal cancer: revising the mutator hypothesis. Cancer Res 61:7369–7374. 11606363

[23] Dellière S, Healey K, Gits-Muselli M, Carrara B, Barbaro A, Guigue N, Lecefel C, Touratier S, Desnos-Ollivier M, Perlin DS, Bretagne S, Alanio A. 2016. Fluconazole and echinocandin resistance of *Candida glabrata* correlates better with antifungal drug exposure rather than with *msh2* mutator genotype in a French cohort of patients harboring low rates of resistance. Front Microbiol 7:2038. doi:10.3389/fmicb.2016.02038. 28066361 PMC5179511

[24] Hou X, Xiao M, Wang H, Yu S-Y, Zhang G, Zhao Y, Xu Y-C. 2018. Profiling of *PDR1* and *MSH2* in *Candida glabrata* bloodstream isolates from a multicenter study in China. Antimicrob Agents Chemother 62:e00153-18. doi:10.1128/AAC.00153-18. 29581110 PMC5971605

[25] Singh A, Healey KR, Yadav P, Upadhyaya G, Sachdeva N, Sarma S, Kumar A, Tarai B, Perlin DS, Chowdhary A. 2018. Absence of azole or echinocandin resistance in *Candida glabrata* isolates in India despite background prevalence of strains with defects in the DNA mismatch repair pathway. Antimicrob Agents Chemother 62:e00195-18. doi:10.1128/AAC.00195-18. 29610199 PMC5971596

[26] Gammie AE, Erdeniz N, Beaver J, Devlin B, Nanji A, Rose MD. 2007. Functional characterization of pathogenic human *MSH2* missense mutations in *Saccharomyces cerevisiae*. Genetics 177:707–721. doi:10.1534/genetics.107.071084. 17720936 PMC2034637

[27] Lang GI, Parsons L, Gammie AE. 2013. Mutation rates, spectra, and genome-wide distribution of spontaneous mutations in mismatch repair deficient yeast. G3 (Bethesda) 3:1453–1465. doi:10.1534/g3.113.006429. 23821616 PMC3755907

[28] Argueso JL, Kijas AW, Sarin S, Heck J, Waase M, Alani E. 2003. Systematic mutagenesis of the *Saccharomyces cerevisiae MLH1* gene reveals distinct roles for Mlh1p in meiotic crossing over and in vegetative and meiotic mismatch repair. Mol Cell Biol 23:873–886. doi:10.1128/MCB.23.3.873-886.2003. 12529393 PMC140715

[29] Demogines A, Wong A, Aquadro C, Alani E. 2008. Incompatibilities involving yeast mismatch repair genes: a role for genetic modifiers and implications for disease penetrance and variation in genomic mutation rates. PLoS Genet 4:e1000103. doi:10.1371/journal.pgen.1000103. 18566663 PMC2413424

[30] Skelly DA, Magwene PM, Meeks B, Murphy HA. 2017. Known mutator alleles do not markedly increase mutation rate in clinical *Saccharomyces cerevisiae* strains. Proc R Soc B 284:20162672. doi:10.1098/rspb.2016.2672. PMC5394658 28404772

[31] Drotschmann K, Shcherbakova PV, Kunkel TA. 2000. Mutator phenotype due to loss of heterozygosity in diploid yeast strains with mutations in *MSH2* and *MLH1*. Toxicol Lett 112–113:239–244. doi:10.1016/S0378-4274(99)00276-3. 10720737

[32] Bui DT, Friedrich A, Al-Sweel N, Liti G, Schacherer J, Aquadro CF, Alani E. 2017. Mismatch repair incompatibilities in diverse yeast populations. Genetics 205:1459–1471. doi:10.1534/genetics.116.199513. 28193730 PMC5378106

[33] Vale-Silva L, Beaudoing E, Tran VDT, Sanglard D. 2017. Comparative genomics of two sequential *Candida glabrata* clinical isolates. G3 (Bethesda) 7:2413–2426. doi:10.1534/g3.117.042887. 28663342 PMC5555451

[34] Serero A, Jubin C, Loeillet S, Legoix-Né P, Nicolas AG. 2014. Mutational landscape of yeast mutator strains. Proc Natl Acad Sci USA 111:1897–1902. doi:10.1073/pnas.1314423111. 24449905 PMC3918763

[35] Tran HT, Keen JD, Kricker M, Resnick MA, Gordenin DA. 1997. Hypermutability of homonucleotide runs in mismatch repair and DNA polymerase proofreading yeast mutants. Mol Cell Biol 17:2859–2865. doi:10.1128/MCB.17.5.2859. 9111358 PMC232138

[36] Greene CN, Jinks-Robertson S. 1997. Frameshift intermediates in homopolymer runs are removed efficiently by yeast mismatch repair proteins. Mol Cell Biol 17:2844–2850. doi:10.1128/MCB.17.5.2844. 9111356 PMC232136

[37] Wierdl M, Dominska M, Petes TD. 1997. Microsatellite instability in yeast: dependence on the length of the microsatellite. Genetics 146:769–779. doi:10.1093/genetics/146.3.769. 9215886 PMC1208050

[38] Kroutil LC, Register K, Bebenek K, Kunkel TA. 1996. Exonucleolytic proofreading during replication of repetitive DNA. Biochemistry 35:1046–1053. doi:10.1021/bi952178h. 8547240

[39] Rhodes J, Beale MA, Vanhove M, Jarvis JN, Kannambath S, Simpson JA, Ryan A, Meintjes G, Harrison TS, Fisher MC, Bicanic T. 2017. A population genomics approach to assessing the genetic basis of within-host microevolution underlying recurrent cryptococcal meningitis infection. G3 (Bethesda) 7:1165–1176. doi:10.1534/g3.116.037499. 28188180 PMC5386865

[40] Thompson DA, Desai MM, Murray AW. 2006. Ploidy controls the success of mutators and nature of mutations during budding yeast evolution. Curr Biol 16:1581–1590. doi:10.1016/j.cub.2006.06.070. 16920619

[41] Raynes Y, Gazzara MR, Sniegowski PD. 2011. Mutator dynamics in sexual and asexual experimental populations of yeast. BMC Evol Biol 11:158. doi:10.1186/1471-2148-11-158. 21649918 PMC3141426

[42] Raynes Y, Gazzara MR, Sniegowski PD. 2012. Contrasting dynamics of a mutator allele in asexual populations of differing size. Evolution 66:2329–2334. doi:10.1111/j.1558-5646.2011.01577.x. 22759305 PMC3389705

[43] Reenan RA, Kolodner RD. 1992. Characterization of insertion mutations in the *Saccharomyces cerevisiae MSH1* and *MSH2* genes: evidence for separate mitochondrial and nuclear functions. Genetics 132:975–985. doi:10.1093/genetics/132.4.975. 1334021 PMC1205253

[44] Williamson MS, Game JC, Fogel S. 1985. Meiotic gene conversion mutants in *Saccharomyces cerevisiae*. I. Isolation and characterization of pms1-1 and pms1-2. Genetics 110:609–646. doi:10.1093/genetics/110.4.609. 3896926 PMC1202584

[45] Selmecki AM, Maruvka YE, Richmond PA, Guillet M, Shoresh N, Sorenson AL, De S, Kishony R, Michor F, Dowell R, Pellman D. 2015. Polyploidy can drive rapid adaptation in yeast. Nature 519:349–352. doi:10.1038/nature14187. 25731168 PMC4497379

[46] Sliwa P, Kluz J, Korona R. 2004. Mutational load and the transition between diploidy and haploidy in experimental populations of the yeast *Saccharomyces cerevisiae*. Genetica 121:285–293. doi:10.1023/b:gene.0000039846.12313.98. 15521427

[47] Tanaka MM, Bergstrom CT, Levin BR. 2003. The evolution of mutator genes in bacterial populations: the roles of environmental change and timing. Genetics 164:843–854. doi:10.1093/genetics/164.3.843. 12871898 PMC1462624

[48] dos Reis TF, Silva LP, de Castro PA, do Carmo RA, Marini MM, da Silveira JF, Ferreira BH, Rodrigues F, Lind AL, Rokas A, Goldman GH. 2019. The *Aspergillus fumigatus* mismatch repair *MSH2* homolog is important for virulence and azole resistance. mSphere 4:e00416-19. doi:10.1128/mSphere.00416-19. 31391280 PMC6686229

[49] Giraud A, Radman M, Matic I, Taddei F. The rise and fall of mutator bacteria. 2001. Curr Opin Microbiol 4:582–585. doi:10.1016/s1369-5274(00)00254-x. 11587936

[50] Desai MM, Fisher DS. 2011. The balance between mutators and nonmutators in asexual populations. Genetics 188:997–1014. doi:10.1534/genetics.111.128116. 21652523 PMC3176104

[51] McDonald MJ, Hsieh Y-Y, Yu Y-H, Chang S-L, Leu J-Y. 2012. The evolution of low mutation rates in experimental mutator populations of *Saccharomyces cerevisiae*. Curr Biol 22:1235–1240. doi:10.1016/j.cub.2012.04.056. 22727704

[52] Brown SM, Campbell LT, Lodge JK. 2007. *Cryptococcus neoformans*, a fungus under stress. Curr Opin Microbiol 10:320–325. doi:10.1016/j.mib.2007.05.014. 17707685 PMC2570326

[53] Legrand M, Chan CL, Jauert PA, Kirkpatrick DT. 2007. Role of DNA mismatch repair and double-strand break repair in genome stability and antifungal drug resistance in *Candida albicans*. Eukaryot Cell 6:2194–2205. doi:10.1128/EC.00299-07. 17965250 PMC2168241

[54] Giraud A, Matic I, Tenaillon O, Clara A, Radman M, Fons M, Taddei F. 2001. Costs and benefits of high mutation rates: adaptive evolution of bacteria in the mouse gut. Science 291:2606–2608. doi:10.1126/science.1056421. 11283373

[55] Dong S, Raffaele S, Kamoun S. 2015. The two-speed genomes of filamentous pathogens: waltz with plants. Curr Opin Genet Dev 35:57–65. doi:10.1016/j.gde.2015.09.001. 26451981

[56] Brent KJ, Hollomon DW. 2007. Fungicide resistance in crop pathogens: how can it be managed? *In* FRAC Monograph 1, vol. 2. CropLife International, Brussels, Belgium.

[57] Chen Y, Zhou M-G. 2009. Characterization of *Fusarium graminearum* isolates resistant to both carbendazim and a new fungicide JS399-19. Phytopathology 99:441–446. doi:10.1094/PHYTO-99-4-0441. 19271986

[58] Ritchie DF. 1983. Mycelial growth, peach fruit-rotting capability, and sporulation of strains of *Monilinia fructicola* resistant to dichloran, iprodione, procymidone, and vinclozolin. Phytopathology 73:44–47. doi:10.1094/Phyto-73-44.

[59] Karaoglanidis GS, Thanassoulopoulos CC, Ioannidis PM. 2001. Fitness of *Cercospora beticola* field isolates–resistant and–sensitive to demethylation inhibitor fungicides. Eur J Plant Pathol 107:337–347. doi:10.1023/A:1011219514343.

[60] Beever RE, Laracy EP, Pak HA. 1989. Strains of *Botrytis cinerea* resistant to dicarboximide and benzimidazole fungicides in New Zealand vineyards. Plant Pathol 38:427–437. doi:10.1111/j.1365-3059.1989.tb02163.x.

[61] Ma L-J, van der Does HC, Borkovich KA, Coleman JJ, Daboussi M-J, Di Pietro AD, Dufresne M, Freitag M, Grabherr M, Henrissat B, Houterman PM, Kang S, Shim W-B, Woloshuk C, Xie X, Xu J-R, Antoniw J, Baker SE, Bluhm BH, Breakspear A, Brown DW, Butchko RAE, Chapman S, Coulson R, Coutinho PM, Danchin EGJ, Diener A, Gale LR, Gardiner DM, Goff S, Hammond-Kosack KE, Hilburn K, Hua-Van A, Jonkers W, Kazan K, Kodira CD, Koehrsen M, Kumar L, Lee Y-H, Li L, Manners JM, Miranda-Saavedra D, Mukherjee M, Park G, Park J, Park S-Y, Proctor RH, Regev A, Ruiz-Roldan MC, Sain D, et al. 2010. Comparative genomics reveals mobile pathogenicity chromosomes in *Fusarium*. Nature 464:367–373. doi:10.1038/nature08850. 20237561 PMC3048781

[62] Milo S, Harari-Misgav R, Hazkani-Covo E, Covo S. 2019. Limited DNA repair gene repertoire in ascomycete yeast revealed by comparative genomics. Genome Biol Evol 11:3409–3423. doi:10.1093/gbe/evz242. 31693105 PMC7145719

[63] Gambhir N, Kamvar ZN, Higgins R, Amaradasa BS, Everhart SE. 2021. Spontaneous and fungicide-induced genomic variation in *Sclerotinia sclerotiorum*. Phytopathology 111:160–169. doi:10.1094/PHYTO-10-20-0471-FI. 33320026

[64] Phillips MA, Steenwyk JL, Shen XX, Rokas A. 2021. Examination of gene loss in the DNA mismatch repair pathway and its mutational consequences in a fungal phylum. Genome Biol Evol 13:evab219. doi:10.1093/gbe/evab219. 34554246 PMC8597960

[65] Jeon J, Choi J, Lee G-W, Dean RA, Lee Y-H. 2013. Experimental evolution reveals genome-wide spectrum and dynamics of mutations in the rice blast fungus, *Magnaporthe oryzae*. PLoS One 8:e65416. doi:10.1371/journal.pone.0065416. 23741492 PMC3669265

[66] Miao J, Mu W, Bi Y, Zhang Y, Zhang S, Song J, Liu X. 2021. Heterokaryotic state of a point mutation (H249Y) in SDHB protein drives the evolution of thifluzamide resistance in *Rhizoctonia solani*. Pest Manag Sci 77:1392–1400. doi:10.1002/ps.6155. 33098218

[67] Covo S. 2020. Genomic instability in fungal plant pathogens. Genes 11:421. doi:10.3390/genes11040421. PMC7230313 32295266

[68] Putnam CD, Kolodner RD. 2017. Pathways and mechanisms that prevent genome instability in *Saccharomyces cerevisiae*. Genetics 206:1187–1225. doi:10.1534/genetics.112.145805. 28684602 PMC5500125

[69] Goldman GH, McGuire SL, Harris SD. 2002. The DNA damage response in filamentous fungi. Fungal Genet Biol 35:183–195. doi:10.1006/fgbi.2002.1344. 11929209

[70] Goldman GH, Kafer E. 2004. *Aspergillus nidulans* as a model system to characterize the DNA damage response in eukaryotes. Fungal Genet Biol 41:428–442. doi:10.1016/j.fgb.2003.12.001. 14998526

